# Effects of influent COD/N ratios on nitrous oxide emission in a sequencing biofilm batch reactor for simultaneous nitrogen and phosphorus removal

**DOI:** 10.1038/s41598-017-06943-0

**Published:** 2017-08-07

**Authors:** Guanghuan Ge, Jianqiang Zhao, Xiaoling Li, Xiaoqian Ding, Aixia Chen, Ying Chen, Bo Hu, Sha Wang

**Affiliations:** 1 School of Environmental Science and Engineering, Chang’an University, Xi’an, China; 2 School of Civil Engineering, Chang’an University, Xi’an, China; 3 Key Laboratory of Subsurface Hydrology and Ecological Effect in Arid Region of Ministry of Education, Xi’an, China

## Abstract

The characteristics of N_2_O emissions from an anaerobic/aerobic/anoxic (A/O/A) sequencing biofilm batch reactor (SBBR) were investigated under different influent COD/nitrogen (C/N) ratios (from 1–4). Results indicated that the C/N ratios affected the quantity of polyhydroxybutyrate (PHB) and residual organic substances after the anaerobic period, resulting in the largest N_2_O emission during aerobic period occurred at a C/N of 2. Moreover, during the anoxic PHB-driven denitrification period, the rapid decline in the dissolved N_2_O concentration indicated that the nitrite inhibition threshold for N_2_O reduction increased with the increased C/N ratios, which means the higher influent C/N ratios could lower the inhibition of nitrite on N_2_O reduction. Finally, more PHB and residual organic substances were provided to denitrification at a high C/N ratio, resulting in less total N_2_O emission was achieved at a high C/N ratio in the A/O/A SBBR.

## Introduction

Stringent sewage discharge standards require the development of biological sewage treatment systems. A sequencing batch biofilm reactor (SBBR) was proposed to realize a compact structure, flexible operation, and highly efficient simultaneous nitrogen and phosphorus removal system^1^. The biofilm system provides different sub-zones for various types of bacteria, allowing each type to find its niche. Additionally, a biofilm system can maximize sludge retention time (SRT). Thus, different bacteria with different SRTs can be developed in a single reactor, including ammonia oxidizing bacteria (AOBs), nitrite oxidizing bacteria (NOBs), phosphorous accumulating organisms (PAOs), denitrifying phosphorous accumulating organisms (DNPAOs) and glycogen accumulating organisms (GAOs)^2^. Simultaneous nitrification denitrification (SND) and phosphorus removal have been previously reported in biofilm systems^3, 4^.

A modified systems operated at an anaerobic/aerobic/anoxic (A/O/A) mode were developed to fully utilize the organics in wastewater for nitrogen and phosphorus removal^2, 5–7^. It was demonstrated that the A/O/A process allows DNPAOs to take an active part in the simultaneous removal of nitrogen and phosphorus in an SBR when a suitable quantity of carbon substrate is supplied at the aerobic conditions^5^. In the A/O/A process, microorganisms were capable of storing the carbon source as polyhydroxybutyrate (PHB) and glycogen, which were the electron donors for endogenous denitritation^7^. According to Yin *et al*.^2^, the A/O/A biofilm system would eliminate the need for external carbon augmentation and could also potentially improve the total nitrogen (TN) removal. The 87.30% ± 11.80% of phosphorus removal efficiency was previously achieved in the A/O/A process proposed by Xu *et al*.^6^. The use of the organic carbon present in the influent wastewater would be maximized to drive simultaneous nitrogen and phosphorus removal in the A/O/A process.

However, detailed off-gas analysis in several studies of the simultaneous nitrogen and phosphorus removal process operated in alternating anaerobic-aerobic mode have shown that nitrous oxide (N_2_O) rather than N_2_ was the major denitrification end-product^8, 9^, which is of significant environmental concern due to the high global warming potential of N_2_O^10^. The N_2_O emission significantly diminishes the overall benefits of the simultaneous nitrogen and phosphorus removal process (e.g., the anaerobic-aerobic system) and limits the prospect of implementing this process in wastewater treatment plants. The nitrogen removal in the A/O/A system was primarily based on nitrification and/or SND in the aerobic period and denitrification in the anoxic period^2, 7^. The N_2_O emission from the A/O/A system is likely to be unavoidable. Therefore, the study of the N_2_O accumulation factors and N_2_O reduction method in the A/O/A process was an important aspect of our research.

Organic carbon is a critical element to achieve successful denitrification and to control the N_2_O emissions. The chemical oxygen demand (COD)/TN (C/N) ratio is critical to evaluate the carbon source availability in wastewater. It is reported that a low C/N ratio can result in N_2_O accumulation^11, 12^. Miao *et al*.^7^ studied the nitrogen removal in an A/O/A SBR system under a C/N ratio from 1–4, and the tests showed the effluent TN was below 10 mg/L at a C/N ratio of 4. However, little information is available regarding the effect of the C/N ratio on the N_2_O emissions in the A/O/A biofilm system. This research provides a detailed investigation on the effects of influent C/N ratios on N_2_O production in the A/O/A SBBR process. It is the first investigation of N_2_O emissions from an A/O/A biofilm system.

## Materials and Methods

### Description of the SBBR

The experiment was carried out in a SBBR with a working volume of 13.00 L, a diameter of 0.20 m, and a height of 0.50 m, and the SBBR was filled with plastic fiber (12% volume). The concentration of the mixed liquor suspended solids (MLSS) was maintained at 3000–3350 mg/L (A certain volume of activated sludge scraped from the biofilm was measured and calculated as the MLSS in SBBR). A constant airflow for aeration was introduced through a fine air diffuser at the bottom of the reactor, and the aeration rate was maintained at 40 L/h (DO concentration was 1.45–2.20 mg/L). A time controller was used to ensure that the SBBR was ran automatically. The temperature of the SBBR was maintained at 30 ± 2 °C using a thermostatic heater in a water bath. The operational pH ranged between 7.00–7.80. A submersible pump was used to keep the solution completely mixed. The reactor was seeding with the activated sludge from the Wastewater Treatment Plant of Chang’an District of Xi’an, China. The SBBR has been in operation steadily in the A/O/A mode for over 1 year.

### Wastewater composition and operation

The synthetic wastewater contained the following: COD (as glucose) at 50–200 mg/L; ammonium (NH_4_^+^-N (as NH_4_HCO_3_)) at 50 mg/L; KH_2_PO_4_ at 44 mg/L; CaCl_2_ at 16 mg/L; NaHCO_3_ at 1500 mg/L; MgSO_4_.7H_2_O at 50 mg/L; and a trace element solution at 1 mL/L^13^.

During the experiment, regarding the effects of influent C/N ratios in an A/O/A SBBR, the NH_4_^+^-N concentration was maintained at 50 mg/L and the COD concentrations were 50, 100, 150 and 200 mg/L, resulting in the influent C/N ratios of 1–4. The A/O/A SBBR was as follows: feeding (10 min), anaerobic reaction (50 min), aerobic reaction (190–270 min), anoxic reaction (380–460 min), and decanting (10 min). The endpoint of the aerobic period was decided by the real-time control method as introduced by Peng *et al*.^14^, and the entire cycle was maintained for 12 h. The exchange volume was 70%, resulting in a hydraulic retention time of 17 h. The sludge falling off the biofilm was removed periodically from the SBBR. The SRT was maintained at approximately 30 d.

### Analytical methods

Ammonia (NH_4_^+^-N), nitrite (NO_2_^−^-N), nitrate (NO_3_^−^-N), COD and MLSS were conducted in accordance with the standard methods^15^. The TN was based on the sum of NH_4_^+^-N, NO_2_^−^-N, NO_3_^−^-N rather than an independent TN test. On-line data were collected by probes for pH, DO, temperature and dissolved N_2_O concentration. The dissolved N_2_O concentration was measured using a N_2_O microsensor (Unisense, Denmark). Glycogen was measured by the anthrone method^16^. The PHB content was determined by UV spectrophotometry according to Law and Slepecky^17^. The nitrite accumulation ratio (NAR) was calculated according to Zeng *et al*.^18^. The efficiency of the simultaneous nitrification and denitrification (E_SND_) was calculated according to Zeng *et al*.^8^.

### N_2_O production and emission

N_2_O generation and emission were determined according to Ge *et al*.^13^, and Hu *et al*.^19^. N_2_O generation (*r*_*g*_; mg N L^−1^sec^−1^; Eq. (1)), accumulation (*r*_*a*_; mg N L^−1^sec^−1^; Eq. (2)) and emission (*r*_*e*_; mg N L^−1^sec^−1^; Eq. (3)) rates were calculated using balances based on on-line N_2_O liquid concentration measurements.

1 $${r}_{g}={r}_{e}+{r}_{a}$$

2 $${r}_{a}=d{c}_{{N}_{2}O}/dt$$

When N_2_O diffused from water to air, the N_2_O emission rate can be calculated by Equation (3).

3 $${r}_{e}=d{c}_{{N}_{2}O}/dt=-{K}_{La{N}_{2}O}\ast ({C}_{{N}_{2}O}-{C}_{S})$$

where: ${K}_{La{N}_{2}O}$ is the volumetric mass transfer coefficient of N_2_O from water to air (sec^−1^); ${C}_{{N}_{2}O}$ is the N_2_O concentration in liquid (mg N L^−1^); *C*_*S*_ is N_2_O saturation concentration in the liquid in equilibrium with air (mg N/L; assumed as 0); *t* is time (sec). Equation (3) was rewritten as

4 $${r}_{e}=d{c}_{{N}_{2}O}/dt=-{K}_{La{N}_{2}O}\ast {C}_{{N}_{2}O}$$

${K}_{La{N}_{2}O}$ was determined by experiment: firstly, reactor that was at the end of settling phase was diluted using distilled water several times to remove the background components; then moderate N_2_O gas was ingested into the liquid of the reactor and the decrease of N_2_O concentrations were measured. The *r*_*e*_ under certain aeration conditions was found by calculation of the N_2_O reduction per second; finally, ${K}_{La{N}_{2}O}$ was obtained by a linear fitting of *r*_*e*_ and ${C}_{{N}_{2}O}$. The total N_2_O production was obtained by the integral calculation of producing rate during reaction time.

## Results and Discussion

### The N_2_O generation and the removals of TN and P in the A/O/A SBBR process

The N_2_O production and the removals of TN and P in the A/O/A SBBR process are shown in Table 1. The N_2_O conversion rate at the influent C/N ratios of 1, 2, 3 and 4 were 30.55 ± 2.04%, 34.13 ± 1.00%, 28.05 ± 1.85% and 7.28 ± 1.04%, respectively. Generally, the N_2_O emissions (7–35%) in this study are much higher than the 4–7% of N_2_O emission from a SBR system by Chen *et al*.^20^, and 1.3–5.5% obtained from the research by Wang *et al*.^21^. The N_2_O generations were reduced by cancelling the anaerobic phase and extending the idle phase in the SBR by Chen *et al*.^20^, and using free nitrous acid based sludge treatment in a nitritation system by Wang *et al*.^21^. In an anaerobic-low DO aerobic process, however, 17–44% of N_2_O emission rate was determined by Chen *et al*.^22^. In this study, the DO concentration was also maintained at a low level to achieve simultaneous nitritation and denitritation during the aerobic period. The low DO condition may stimulate the high N_2_O production in this paper.

**Table 1:** Effects of C/N ratio on N_2_O generation and the removals of TN and P from synthetic wastewater treated by the A/O/A SBBR process.

	C/N^a^:1	C/N:2	C/N:3	C/N:4
N_2_O conversion rate^b^ (%)	30.55 ± 2.04	34.13 ± 1.00	28.05 ± 1.85	7.28 ± 1.04
TN removal efficiency (%)	82.46 ± 1.65	91.75 ± 5.60	97.49 ± 2.67	98.30 ± 2.53
P removal efficiency (%)	6.35 ± 8.22	9.07 ± 7.20	82.79 ± 1.87	27.44 ± 6.21
Average NAR^c^ (%)	58.56	73.90	86.19	73.43
Average E_SND_^d^ (%)	27.61	40.62	53.15	78.61
Average Ammonia oxidation rate (mg/L.min)	0.13	0.13	0.11	0.11

^a^C/N = COD/influent total nitrogen; ^b^N_2_O-N conversion rate = (total N_2_O-N production)/(total nitrogen removed) × 100%; ^c^NAR: nitrite accumulation ratio; ^d^E_SND_: the efficiency of simultaneous nitrification and denitrification.

The N_2_O conversion rates decreased with the increased C/N ratios except at C/N ratio of 1. The largest N_2_O conversion rate occurred at C/N ratio of 2. The reactions under different influent C/N ratios were operated at 12 h-cycles. The lowest TN removal efficiency (82.46 ± 2.04%) occurred at C/N ratio of 1. Part of NO_x_^−^-N was not denitrified completely and was discharged in effluent, resulting in the N_2_O conversion rate at C/N of 1 was lower than that at C/N of 2. The N_2_O conversion rate under influent C/N ratio of 4 was much lower than that at the influent C/N ratios of 1–3. It indicates that adequate carbon source was beneficial to control the N_2_O emission in this A/O/A biofilm process.

In the A/O/A SBBR, the carbon source in the influent was converted to PHB during the anaerobic period and the denitrification rate during aeration (the E_SND_) was affected by the PHB as the electron donor. As shown in Table 1, the E_SND_ values of the influent C/N ratios of 1, 2, 3 and 4 were 27.61%, 40.62%, 53.15% and 78.61%, respectively, which increased with the increased C/N ratios. For the influent C/N ratios of 2 and 3, the post-denitrification driven by internal carbon source contributed to the advanced nitrogen removal efficiencies of 91.75 ± 5.60–97.49 ± 2.67% in the A/O/A SBBR, while with abundant organic carbon sources at the C/N ratio of 4, TN was primarily removed via the SND during aerobic period with the SND efficiency of 78.61% in the A/O/A SBBR.

The changes of phosphorus concentrations under different C/N ratios are shown in Fig. S1 (the supplementary information). As shown in Fig. SI, for the influent C/N ratios of 1 and 2, the performances of anaerobic phosphorus release, aerobic phosphorus uptake and denitrifying phosphorus removal did not occur, implying that the low influent C/N ratio adversely affected the phosphorus removal in the A/O/A SBBR. The carbon in the influent with C/N ratios of 1–2 was too low, and the residual NOx^−^-N (NO_2_^−^-N and/or NO_3_^−^-N) from the previous cycle was still present in the current cycle. Thus, the advanced phosphorus removal failed to occur when the NO_X_^−^-N was present in the designated anaerobic zone due to the competition for carbon between the denitrifying organisms and the PAOs. Meanwhile, nitrite accumulation is known to inhibit phosphorus uptake, which may cause damage to the polyphosphate kinases or PHB oxidation^23^.

The typical cycle profile of the A/O/A SBBR at the C/N ratio of 4 is shown in Fig. S2 (the supplementary information). As shown in Fig. S2, during the anoxic period of the 5.0th–7.5th h, the NO_2_^−^-N concentration decreased with no phosphorus release. The NO_2_^−^-N concentration decreased to a minimum value at the 7.5th h of the anoxic period when phosphorus release began. Thus, the untimely depletion of NO_3_^−^-N/NO_2_^−^-N during the anoxic period then caused secondary P release^24^. The secondary phosphorus release under the influent C/N ratio of 4 would be avoided by reasonable adjustments the duration of the post anoxic period (e.g., the anoxic period duration was adjusted to 2.5 h) (Fig. S2). This indicates the reason of the secondary phosphorus release was that the duration of the anoxic period rather than the high influent C/N ratio. The advanced phosphorus removal was achieved at the influent C/N ratio of 3 in the A/O/A SBBR, with the phosphorus removal efficiency of 82.79 ± 1.87%. In total, the greater nitrogen removal performance appeared under higher influent C/N ratios (3–4), while the high efficiency of the phosphorus removal did not appear simultaneously at the C/N ratio of 4 in the A/O/A SBBR.

One notable point that should be discussed here is the presence of glucose in the wastewater treated in this study. A large number of publications have demonstrated that the presence of glucose in wastewater readily stimulated the proliferation of GAO in reactor, which thereby caused deterioration or even failure in biological phosphorus removal^25–27^. Apparently, the results obtained in this study were inconsistent with those reported previously. Several researchers also found good P-removal in glucose-fed reactors^28–30^. Chen *et al*. (2002)^31^ even revealed that the presence of glucose promoted the phosphorus removal efficiency. This fact suggested that glycogen may replace PHA to provide energy for PAO to take up phosphorus in this A/O/A SBBR, which was different from the conventionally metabolic behavior. From the perspective of microorganisms, Nakamura *et al*.^32^ observed that *Microlunatus phosphovorus* can uptake glucose and release phosphorus under anaerobic conditions, with subsequent uptake of phosphorus under aerobic conditions. Thus, *Microlunatus phosphovorus* might be a possible PAO in the presence of glucose.

### The typical cycle results of the A/O/A SBBR at the C/N ratio of 3

**Figure 1:**
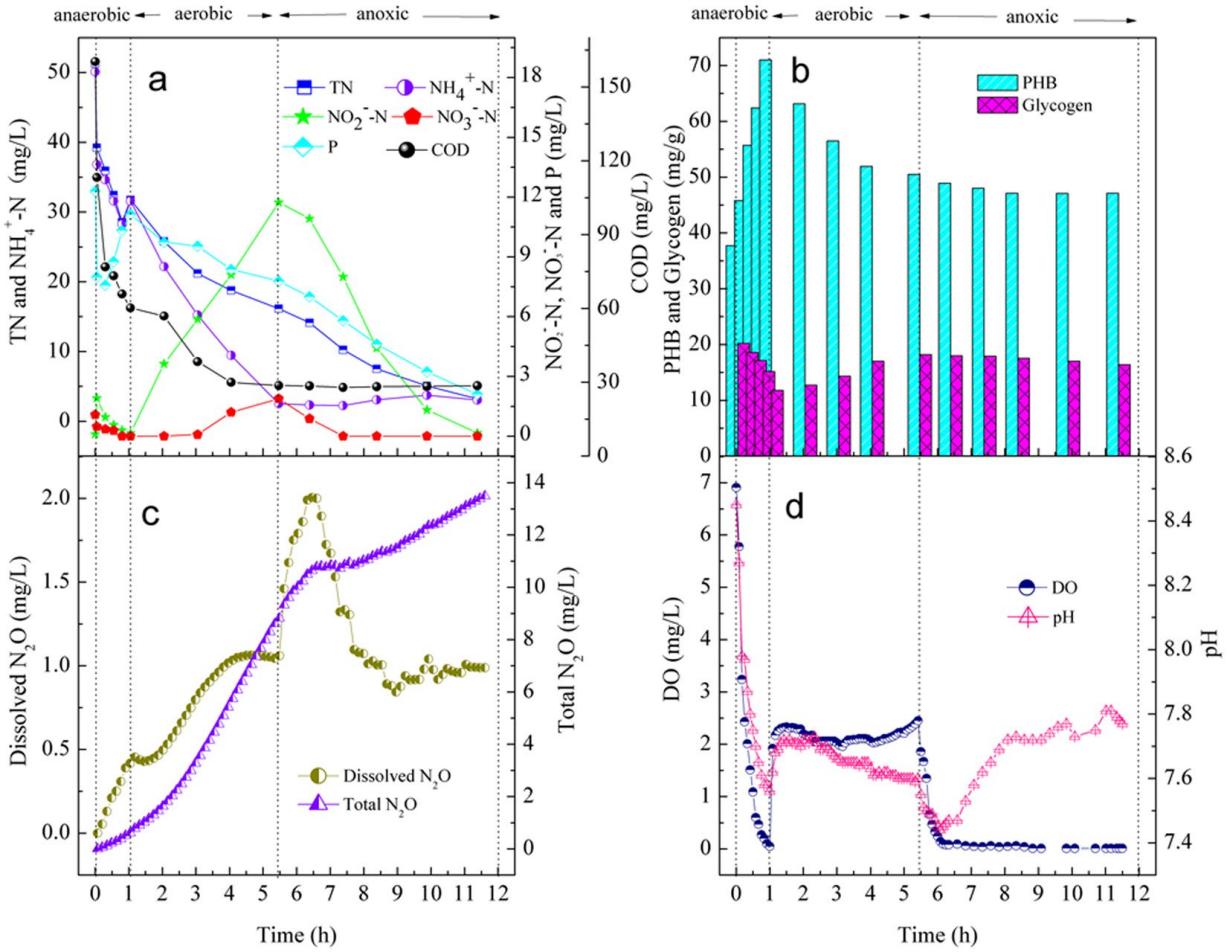
Typical profiles of nitrogen compounds and control parameters in the A/O/A SBBR at influent C/N ratio of 3.

To better understand the N_2_O emissions as well as nitrogen and phosphorus removal pathways, the typical cycle profile of the A/O/A SBBR at the C/N ratio of 3 is shown in Fig. 1. During the anaerobic period (50 min), the consumption of the carbon source and the glycogen associated with PHB accumulation and phosphorus release indicated that the anaerobic condition was conducive for microorganisms (e.g., PAOs and GAOs) to store the carbon source. These microorganisms could obtain energy and reduce power from the glycogen degradation via polyphosphate hydrolysis and/or glycolysis for the synthesis of the PHB^33^. Only 0.61 mg/L N_2_O was emitted in the anaerobic periods.

At the aerobic period with a 40 L/h aeration rate, the DO concentration of the reactor at the influent C/N ratio of 3 was 2.14 ± 0.10 mg/L (Fig. 1(d)). The 11.73 mg/L NO_2_^−^-N was accumulated at the end of aerobic period. The 15.60 mg/L TN was removed and 3.35 mg/L of phosphorus was absorbed during the aeration. The N_2_O production during aerobic period was 5.46 mg/L. During the aerobic period in the A/O/A SBBR, both the AOB denitrification and hydroxylamine (NH_2_OH) oxidation pathways contributed to N_2_O production^34, 35^. In AOB denitrification, NH_4_^+^-N is converted by nitrifiers to NO_2_^−^-N followed by the reduction of NO_2_^−^-N to N_2_O etc.^36^ Peng *et al*.^35^ studied the N_2_O production by AOB under different DO concentrations, and the results showed that the contribution of AOB denitrification were 66–95%, accompanied by a corresponding increase in the contribution by the NH_2_OH oxidation pathway when DO increased from 0.2 to 3.0 mg O_2_/L. In addition, in the A/O/A SBBR system in this paper, the DO concentrations in the inner biofilm were relatively lower than that in liquid due to the mass transfer resistance of biofilm on the DO. The heterotrophic denitrification process could carry out in the inner biofilm during the aeration using the remainder carbon or internal storage carbon substance. von Schulthess *et al*.^37^, reported that N_2_O accumulation would occur when the activity of N_2_O reductase was inhibited by the presence of oxygen in the heterotrophic denitrification.

A large portion of the NO_x_^−^-N (11.73 mg/L) was removed with the phosphorus uptake (5.68 mg/L) during the post anoxic period in the A/O/A SBBR. The secondary phosphorus release did not occur during the cycle of the influent C/N ratio of 3 because NO_2_^−^-N was denitrified during the entire process and no untimely depletion of NO_2_^−^-N occurred. The COD maintained at a low level (28.68 mg/L) after the aerobic period. The NO_x_^−^-N concentration decreased with a small change of the COD concentration during the anoxic period (Fig. 1(a)), indicating that the carbon source for denitrification and phosphate uptake under anoxic conditions was not external but internal in the SBBR. The DNPAOs showed the capability of using NO_x_^−^-N as an electron acceptor for denitrification and phosphate uptake^38^, with the internal components (PHB and glycogen) possibly supplying the energy for denitrification.

During the anoxic phase, N_2_O continued to increase as PHB decreased until the NO_2_^−^-N declined to 10.92 mg/L at 6.5 h of reaction. The pH increased until the TN decreased to the lowest level at 11 h of reaction. PHB was consumed as the carbon source for denitrification^9^. In total, 11.73 mg/L NO_2_^−^-N was removed by the PHB-driven denitritation, and 7.42 mg/L N_2_O was produced during the anoxic period. Sixty-three percent of the NO_2_^−^-N was converted to N_2_O. Some studies proposed that the PHB-driven denitrification caused a larger N_2_O production^9, 34, 39^. According to the study of Kampschreur *et al*.^34^, the reason was that the slow degradation of PHB led to the electrons competition among different denitrifying enzymes; and the low nitrous oxide reductase activity and its weakest electronic competition ability caused N_2_O accumulation.

### Effects of C/N ratios on COD consumption and PHB in SBBR

**Figure 2:**
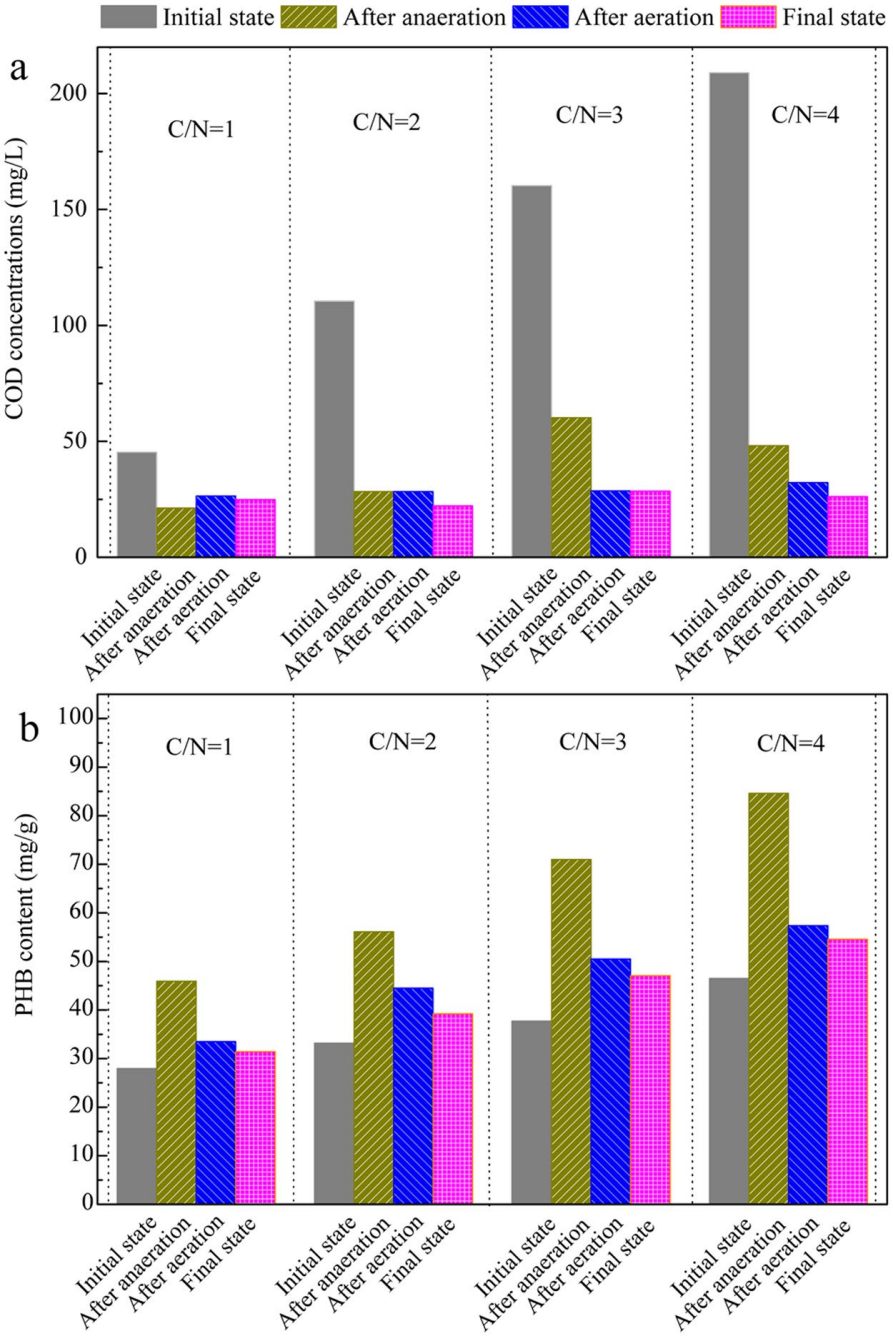
The effects of C/N ratios on the COD consumption (**a**) and PHB (**b**) in the A/O/A SBBR.

The changes in the COD concentrations and PHB contents in the A/O/A SBBR system are shown in Fig. 2. The addition of the anaerobic period after the feeding was beneficial to store the carbon sources^33^. During the anaerobic period, COD was decreased and this was accompanied by the synthesis of PHB. At low C/N ratio of 1 with limited organic substances, a small amount of PHB (17.98 mg/g) was stored in the anaerobic phase (Fig. 2(b)). With the increase of the influent C/N ratios to 3 and 4, the stored PHB increased to 33.28 mg/g and 38.10 mg/g (Fig. 2(b)), respectively. During the aerobic period, the COD decrease means the COD consumption occurred when the C/N ratios at 3 and 4; while, when the C/N ratios at 1 and 2, the COD concentrations decreased to the lowest value at the end of the anaerobic period and the COD consumption during the aeration did not appear in these conditions. The PHB consumption in aerobic period and anoxic period at the influent C/N ratios of 1 was 14.48 mg/g, and increased to 30.02 mg/g at the influent C/N ratio of 4 (Fig. 2(b)). In total, the amounts of the PHB accumulation during the anaerobic period and the PHB consumption in aerobic/anoxic period increased with the increased C/N ratios.

### Effects of C/N ratios on N_2_O emission during the aerobic period

As shown in Table 2, the influent C/N ratio had a significant effect on N_2_O production in the A/O/A SBBR process. During the anaerobic period, there was only a very small amount of the N_2_O produced at the influent C/N ratios of 1–4 due to the sufficient carbon source in the initial stage of the operation and the limited NO_x_^−^-N in the liquid. The N_2_O productions during the aeration at the influent C/N ratios of 1, 2, 3 and 4 were 6.61 ± 1.10, 10.65 ± 1.34, 5.46 ± 0.85 and 2.66 ± 0.05 mg/L, respectively. The result showed that the N_2_O produced at the C/N ratio of 2 was nearly 1.6 times than that at the C/N ratio of 1, while the N_2_O production during the aeration was approximately reduced by 50% when the C/N ratio increased from 2 to 3 and from 3 to 4.

**Table 2:** The effects of influent C/N ratio on N_2_O production and nitrogen conversion in the A/O/A SBBR system.

	C/N^a^ = 1	C/N = 2	C/N = 3	C/N = 4
N_2_O in anaerobic period (mg/L)	0.95 ± 0.05	0.10 ± 0.00	0.61 ± 0.03	0.03 ± 0.01
N_2_O in aerobic period (mg/L)	6.61 ± 1.10	10.65 ± 1.34	5.46 ± 0.85	2.66 ± 0.05
N_2_O in anoxic period (mg/L)	5.43 ± 0.85	5.96 ± 0.65	7.42 ± 1.10	0.92 ± 0.02
Total N_2_O production (mg/L)	12.99 ± 2.20	16.71 ± 1.85	13.49 ± 2.03	3.61 ± 0.15
N_2_O conversion rate^b^	30.55 ± 2.04	34.13 ± 1.00	28.05 ± 1.85	7.28 ± 1.04

^a^C/N = COD/influent total nitrogen; ^b^N_2_O-N conversion rate = (total N_2_O-N production)/(total nitrogen removed) × 100%.

According to the analysis in previous section, during the aeration in the system, the N_2_O can be produced through the AOB denitrification pathway, the aerobic NH_2_OH oxidation pathway in nitrification, and the heterotrophic denitrification. According to Fig. 2, the COD concentration after the anaerobic period was 48–60 mg/L and the decreasing trends occurred during the aerobic period at C/N ratios of 3. This phenomenon demonstrated that the residual biodegradable organic matter in the liquid and/or biofilm after the anaerobic period occurred with the increasing influent C/N ratios. Thus, the heterotrophic denitrification at higher influent C/N ratios (when the C/N ratios were 3 and 4) included the residual biodegradable organic matter-driven denitrification and the PHB-driven denitrification; while, when the C/N ratios were 1 and 2, no COD consumption during the aeration indicates that the heterotrophic denitrification during the aeration was only the PHB-driven denitrification.

Under the influent C/N ratio of 1, the limited available carbon source accompanied limited storage of PHB, resulting in low efficiency of the PHB-driven heterotrophic denitrification and low N_2_O production via PHB-driven denitrification. When the C/N ratio increased from 1 to 2, the storage of PHB increased with the increased carbon source, and the N_2_O production in PHB-driven denitrification during the aerobic period increased with the strengthening denitrification. As Table 1, the average ammonia oxidation rates at C/N ratios of 1 and 2 were 0.13 mg/L.min, it indicates there was little difference between the amounts of N_2_O production via NH_2_OH oxidation at the C/N ratios of 1 and 2. The SND efficiency at the C/N ratio of 2 was higher than that at C/N ratio of 1, mainly due to the more NO_x_^−^-N was denitrified via PHB-driven denitrification at the C/N ratio of 2. Therefore, it could be deduced that the portion of the N_2_O emitted at the influent C/N ratio of 2 was greater than that at the influent C/N ratio of 1 and may be a product of the PHB-driven denitrification.

The more carbon that was involved in the influent, the more biodegradable organic matter after the anaerobic period would be remained in system. The residual biodegradable organic matter-driven denitrification was improved when the influent C/N ratios increased to 3 and 4, leading to a portion of N_2_O reducing to N_2_. Moreover, the amount of NO_2_^−^-N decreased with the improved biodegradable organic matter-driven denitrification, which resulted in less N_2_O production via the nitrifier denitrification at higher C/N ratio conditions. Therefore, the N_2_O production decreased 50% when the C/N ratio increased from 3 to 4, and the quantity of N_2_O produced at the influent C/N ratios of 3 and 4 were less than that at the influent C/N ratio of 2 during the aerobic period.

In the A/O/A SBBR system, the influent C/N ratio affects the cyclic nitrite accumulation level, which may be the major reason for the different N_2_O production. Nitrite level significantly affects N_2_O production from both nitrifier denitrification pathway and denitrifier denitrification pathway. However, the heterotrophic denitrification could be partially inhibited due to the limited carbon source in the aerobic stage under low C/N ratios. And the heterotrophic denitrification was also limited by the presence of oxygen during the aerobic period. Jia *et al*.^40^, reported that the AOB denitrification pathway rather than the heterotrophic denitrification pathway represented the dominant source of N_2_O production in SND process. According to the modeling results of Ding *et al*.^41^, the N_2_O emission from the A/O/A SBR was primarily generated in the aerobic stage by the AOB denitrification pathway (67.84–81.64%), incomplete NH_2_OH oxidation (15.61–32.17%) and heterotrophic denitrification on intracellular polymers (0–12.47%). In total, nitrifier denitrification pathway may be the reason for the higher N_2_O emission at lower C/N ratios conditions.

### Effects of C/N ratios on N_2_O emission during the anoxic period

**Figure 3:**
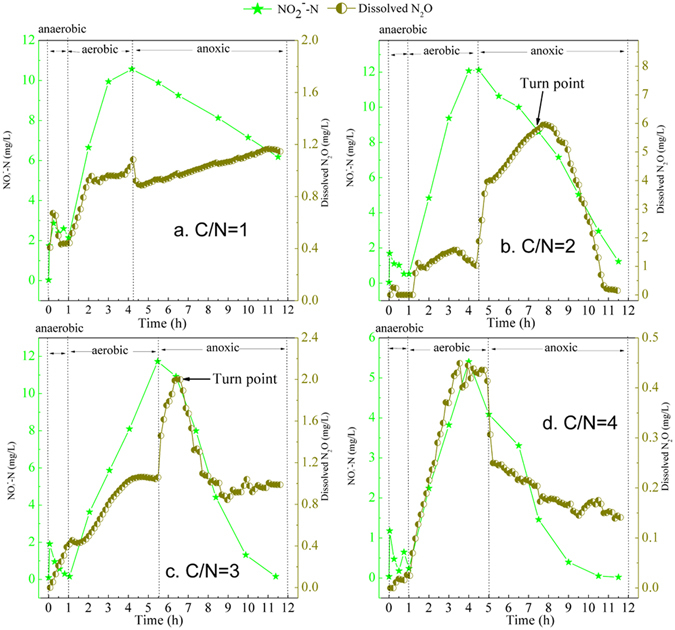
Typical profiles of nitrite and dissolved N_2_O concentrations in the A/O/A SBBR at different influent C/N ratios.

The NO_2_^−^-N accumulation after the aerobic period of the influent C/N ratios of 1, 2, 3 and 4 were 10.57 mg/L, 12.12 mg/L, 11.73 mg/L and 4.09 mg/L, respectively (Fig. 3). During the anoxic period, the rapid decline of the dissolved N_2_O appeared after 3.5 h at the C/N ratio of 2 and at 1.0 h at the C/N ratio of 3 (Fig. 3(b and c)). The phenomenon of the rapid decline of the dissolved N_2_O did not occur at the end of the anoxic period under the C/N ratio of 1 (Fig. 3(a)), but it appeared before the start of the anoxic period under the C/N ratio of 4 (Fig. 3(d)).

Nitrite has generally been recognized as an inhibitor of N_2_O reduction during denitrification^42^. The dissolved N_2_O was further denitrified, leading to the rapid decline of dissolved N_2_O concentration, it indicates the end of the inhibition of NO_2_^−^-N on N_2_O reduction. The results show the nitrite inhibition end time for N_2_O reduction decreased with the increased C/N ratios. Moreover, the NO_2_^−^-N concentrations of the inhibition also varied under different C/N ratios. The inhibition end occurred when the NO_2_^−^-N concentrations were 7.88 mg/L at a C/N ratio of 2 and 10.92 mg/L at C/N ratio of 3. N_2_O was still inhibited when the NO_2_^−^-N concentration declined to 6.18 mg/L at the C/N ratio of 1. Itokawa *et al*.^43^ reported nitrite accumulation (10 mg NO_2_^−^-N/L) as a possible cause of N_2_O production in a denitrifying sludge with an influent COD/N ratio less than 3.5. However, it could be concluded in this paper that the nitrite inhibition threshold for N_2_O reduction increased with increased C/N ratios. The amount of PHB increased with the increasing influent C/N ratio, which may temper the competition for carbon between the denitrifying organisms so that the inhibition of NO_2_^−^-N on N_2_O reduction occurred at a higher nitrite concentration. In total, the higher influent C/N ratio could lower the inhibition of NO_2_^−^-N on N_2_O reduction so that less N_2_O emission could be achieved.

### Effects of C/N ratios on total N_2_O emission

As shown in Table 2, the N_2_O conversion rate at the influent C/N ratio of 4 was 7.28 ± 1.04%, which was much lower than that at the influent C/N ratios of 1–3 (26–35%). The possible explanations for higher N_2_O production in low influent C/N ratios are as follows: (1) the carbon deficiency caused low efficiency of the SND and more accumulation of NO_2_^−^-N. The NO_2_^−^-N stimulated N_2_O production based on the nitrifier denitrification during aerobic period^44, 45^. (2) The inhibitory effect of NO_2_^−^-N on N_2_O reduction caused more N_2_O accumulation via heterotrophic denitrification. (3) A decrease in the rate of PHB availability with the decrease of the influent C/N ratios (Fig. 2) resulted in a limited electron donor for denitrification and less opportunity of N_2_O reductase competition to electronics, leading to the increased N_2_O production under the lower influent C/N ratios. These results demonstrated that the limited availability of electron donors resulted in NO_2_^−^-N accumulation which led to high N_2_O production at lower C/N ratio conditions. Hence, to reduce the production of N_2_O, it is important to control the influent C/N ratio at an optimal value to avoid nitrite accumulation.

In conclusion, we studied the impacts of the C/N ratios on N_2_O emissions in the A/O/A SBBR for nitrogen and phosphorus removal. The lowest N_2_O production was achieved at the C/N ratio of 4. The increased PHB synthesis and residual organic substrates after the anaerobic period resulted in lower N_2_O emissions at a higher C/N ratio. Moreover, the higher influent C/N ratio could lower the inhibition of NO_2_^−^-N on N_2_O reduction, which also led to less N_2_O emission at a higher C/N ratio.

## Electronic supplementary material

Supplementary information
