# Author Correction: Effects of influent COD/N ratios on nitrous oxide emission in a sequencing biofilm batch reactor for simultaneous nitrogen and phosphorus removal

**DOI:** 10.1038/s41598-018-36019-6

**Published:** 2018-11-30

**Authors:** Guanghuan Ge, Jianqiang Zhao, Xiaoling Li, Xiaoqian Ding, Aixia Chen, Ying Chen, Bo Hu, Sha Wang

**Affiliations:** 1 School of Environmental Science and Engineering, Chang’an University, Xi’an, China; 2 School of Civil Engineering, Chang’an University, Xi’an, China; 3 Key Laboratory of Subsurface Hydrology and Ecological Effect in Arid Region of Ministry of Education, Xi’an, China

Correction to: *Scientific Reports* 10.1038/s41598-017-06943-0, published online 07 August 2017

This Article contains errors in the Acknowledgements section.

“This work was supported by the Fundamental Research Funds for the Central Universities (Grant No. 310829165020) and the Shaanxi Province Science and Technology Development Program (Grant No. 2014K15–03–02).”

should read:

“This work was supported by the Fundamental Research Funds for the Central Universities (Grant No. 310829165020) and the National Natural Science Foundation of China (Grant No. 51778057)”

