# Supplementary material for: Effects of influent COD/N ratios on nitrous oxide emission in a sequencing biofilm batch reactor for simultaneous nitrogen and phosphorus removal
Source: Sci Rep. 2017 Aug 7;7:7417. doi: 10.1038/s41598-017-06943-0 (PMC5547147; doi:10.1038/s41598-017-06943-0)
Supplement: Supplementary file 1 — Supplementary information [file 41598_2017_6943_MOESM1_ESM.doc]

**Effects of influent COD/N ratios on nitrous oxide emission in a sequencing biofilm batch reactor for simultaneous nitrogen and phosphorus removal**

Guanghuan Ge^1^, Jianqiang Zhao^1,3,*^, Xiaoling Li^2^, Xiaoqian Ding^1^, Aixia Chen^1,3^,Ying Chen^1^, Bo Hu^2^, Sha Wang^1^

1. School of Environmental Science and Engineering, Chang’an University, Xi’an, China.

2. School of Civil Engineering, Chang’an University, Xi’an, China.

3. Key Laboratory of Subsurface Hydrology and Ecological Effect in Arid Region of Ministry of Education, Xi'an, China.

The changes of phosphorus concentrations in the A/O/A SBBR at influent C/N ratios of 1-4 are shown in **Fig. S1**. **Fig. S2** shows the typical profiles of nitrogen compounds and control parameters in the A/O/A SBBR at influent C/N ratio of 4.


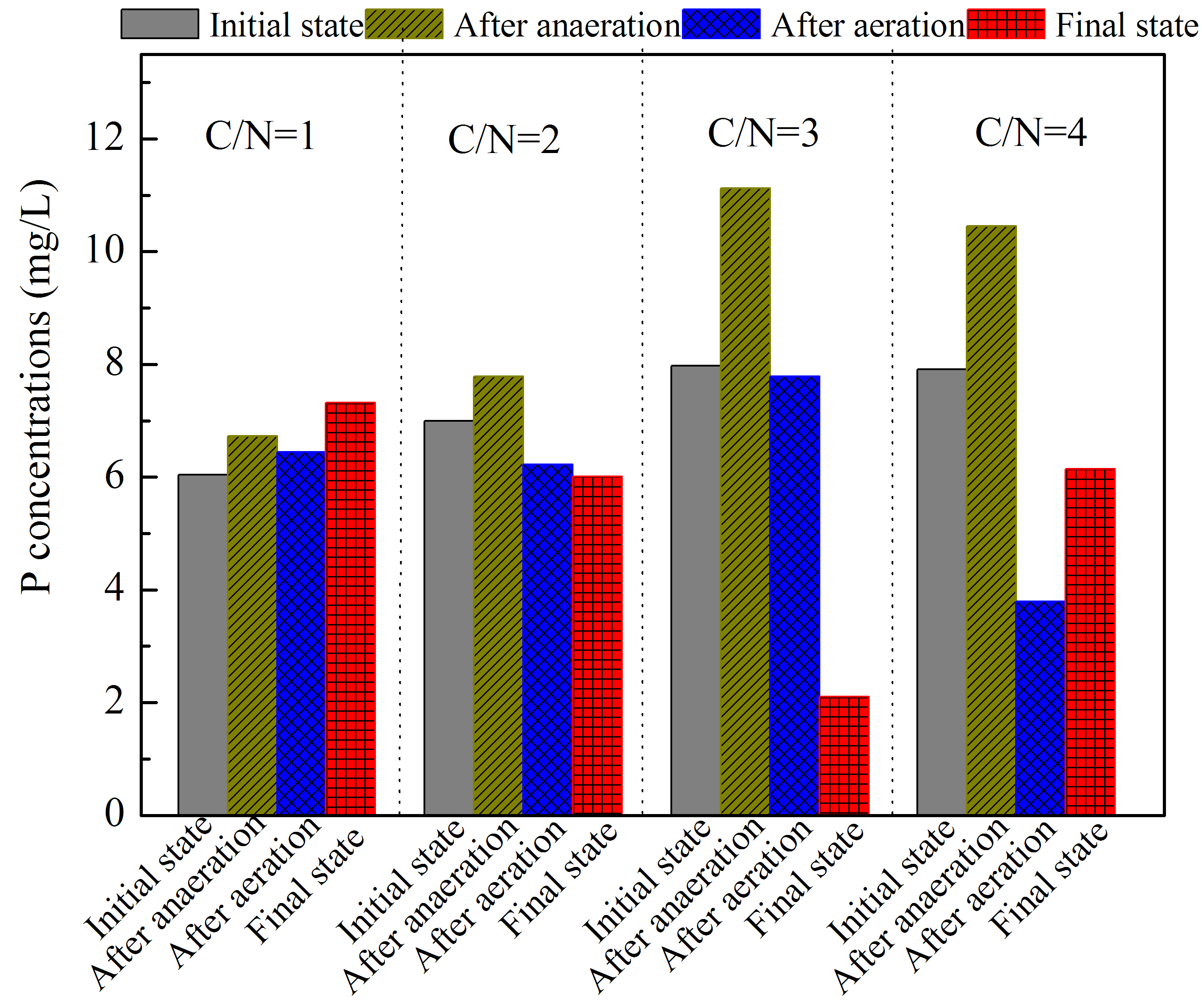


**Fig. S1** The changes of phosphorus concentrations in the A/O/A SBBR at different influent C/N ratios.


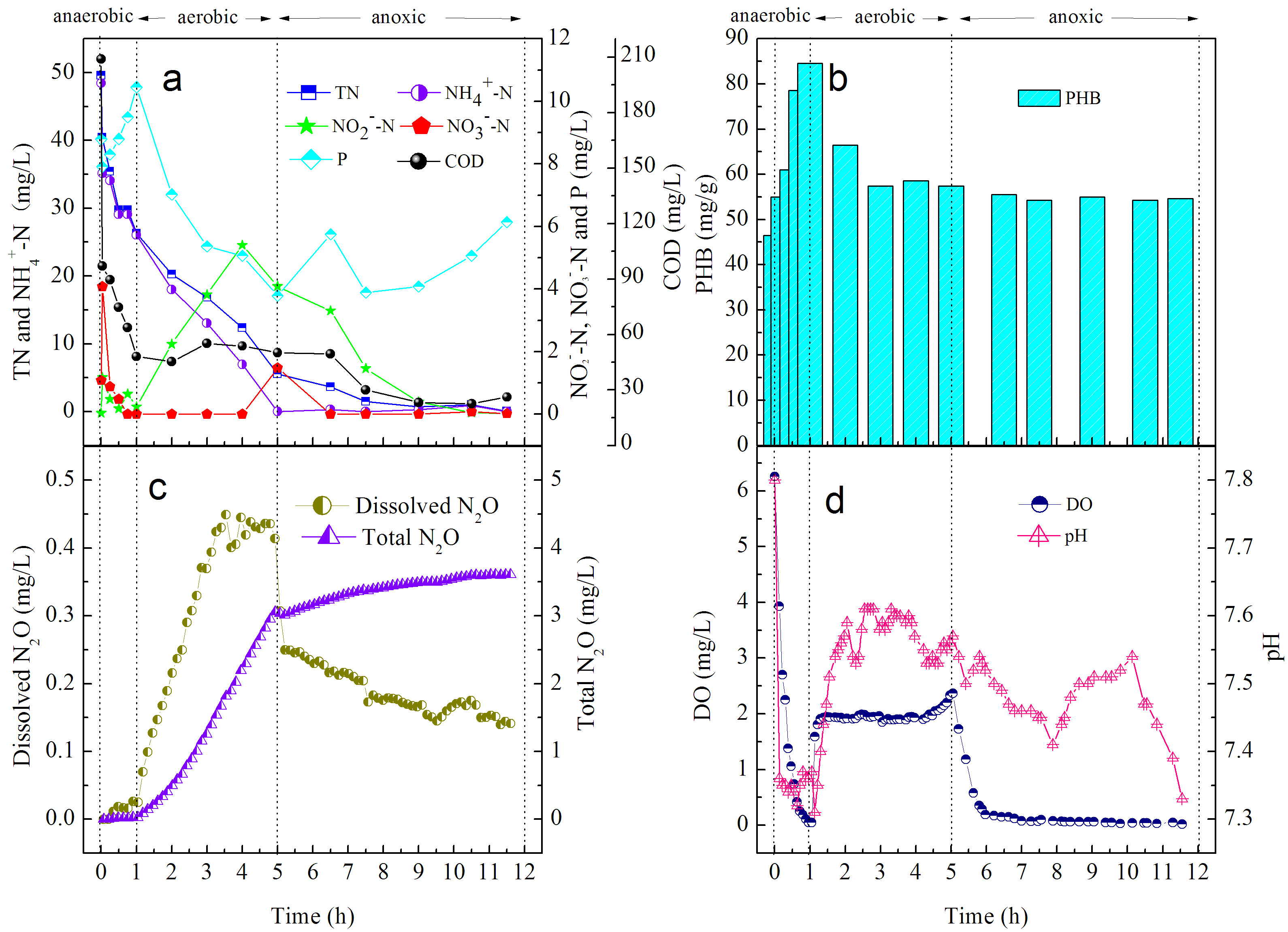


**Fig. S2** Typical profiles of nitrogen compounds and control parameters in the A/O/A SBBR at influent C/N ratio of 4.
